# The application of Rasch measurement theory to psychiatric clinical outcomes research: Commentary on … Screening for depression in primary care†

**DOI:** 10.1192/pb.bp.115.052290

**Published:** 2016-10

**Authors:** Skye P. Barbic, Stefan J. Cano

**Affiliations:** 1 University of British Columbia, Vancouver, Canada; 2 Modus Outcomes, Stotfold, UK

## Abstract

This commentary argues the importance of robust, meaningful assessment of clinical and functional outcomes in psychiatry. Outcome assessments should be fit for the purpose of measuring relevant concepts of interest in specific clinical settings. As well, the measurement model selected to develop and test assessments can be critical for guiding care. Three types of measurement models are presented: classical test theory, item response theory, and Rasch measurement theory. To optimise current diagnostic and treatment practices in psychiatry, careful consideration of these models is warranted..

Unlike many fields in medicine, most clinical outcomes in psychiatry are not directly observable and cannot be captured with diagnostic tests such as blood work or imaging. In recent years, the importance of the routine use of clinical outcome assessments (patient-reported outcomes, clinician-reported outcomes, observer-reported outcomes and performance outcomes) for measuring the symptoms of disease and treatment outcomes has been increasingly emphasised.^1^ Clinical outcome assessments such as the Patient Health Questionnaire-9 (PHQ-9)^2^ are now commonly used in clinical research and practice to provide an assessment of a patient's severity of mood and improvement in response to treatment.^3^ More broadly, as the demand increases for a broad range of mental health services to be patient-centred, clinical outcome assessments are used to capture outcomes such as sustained symptom reduction, return to full functioning and optimal patient well-being.^4^

To optimise mental healthcare, clinical outcome assessments used in psychiatry should be shown to be fit for purpose. They should appropriately capture the concept of interest (e.g. depression) in the context of use (e.g. patients attending primary care clinics reporting symptoms of depression).^1^ They should also be underpinned by an appropriate measurement model, that is they should have evidence that the summed score of their individual items is ‘psychometrically sound’.^1^ To this end, there are three main psychometric approaches based on three types of measurement model: classical test theory (CTT), Rasch measurement theory (RMT) and item response theory (IRT).^5^

The current dominant paradigm in clinical outcomes research is CTT, the foundations of which were laid down by Charles Spearman at the turn of the twentieth century.^6^ CTT is associated with the psychometric properties most commonly recognised and understood by clinicians (e.g. reliability, validity and ability to detect change). However, there are four important limitations^7^ that prevent CTT methodology from fulfilling the requirements of scientific rigour demanded of high-stakes clinical decision-making: (a) measurements generated are ordinal rather than interval; (b) scores for persons and samples are scale dependent; (c) scale properties, such as reliability and validity, are sample dependent; (d) data can support group-level inferences but are not suitable for individual patient measurement.

Georg Rasch, a Danish mathematician, argued that the core requirement of social measurement should be the same as that in physical measurement, and developed the simple logistic model now known as the ‘Rasch model’.^8^ In essence, RMT methods assess the extent to which observed clinical outcome assessment data (e.g. patient ratings on the items of the PHQ-9) ‘fit’ with predictions of those ratings from the Rasch model (which defines how a set of items should perform to generate reliable and valid measurements).^8^ The difference between the expected and observed scores reveals the extent to which valid measurement is achieved. In turn, this gives rise to a range of potential investigations to better understand the extent to which the clinical outcome assessment under investigation is an appropriate measurement instrument (e.g. scale-to-sample targeting, adequacy of type and kind of response options, item and person fit, item dependency (or bias), stability between subgroups).^7,9^ Importantly, RMT addresses^7^ each of the four limitations of CTT described above: (a) linear measurements can be constructed from ordinal-level data; (b) item estimates provided are free from the sample distribution and person estimates are free from the scale distribution; (c) subsets of items from each scale rather than all items can be used (i.e. the foundation for item banking and computerised adaptive testing); (d) estimates are suitable for individual person analyses rather than only for group comparison studies.

IRT is another body of psychometric methodology that is used to ascertain the degree to which a given model and parameter estimates can account for the structure of and statistical patterns in a clinical outcome assessment dataset.^10^ The distinction between RMT and IRT is subtle but important. IRT models are statistical models used to explain data, and the aim of an IRT analysis is to find the statistical model that best explains the observed data.^9^ By contrast, the aim of RMT is to determine the extent to which observed clinical outcome assessment data satisfy the measurement model.^8^ When the data do not fit the model, they are examined to try to explain the misfit. This is the central tenet of the Rasch model and one that distinguishes it from IRT models. Specifically, its defining property is its mathematical embodiment of the principle of invariant comparison. Thus, the comparison of two people is independent of which items are used within a set of items assessing the same concept of interest. In this way, the Rasch model is taken as a criterion for the structure of the responses, rather than simply a statistical description of the responses from patients. This central tenet distinguishes the RMT diagnostic paradigm from the IRT modelling paradigm.^9^

In this issue, Horton and Perry provide an example of diagnostic information that can be attained using RMT methods, not available using information gleaned from CTT or IRT methods.^11^ The availability and increased application of RMT psychometric methods for developing and evaluating clinical outcome assessments in psychiatry has important implications for future research and practice. By better understanding the strengths, weaknesses and measurement potential of such assessments, we are able to build an evidence base towards optimising the organisation and delivery of healthcare in psychiatry.^12^
